# Comparative Cardiovascular Outcomes of Dapagliflozin Versus Empagliflozin in Patients With Type 2 Diabetes: A Meta-Analysis

**DOI:** 10.7759/cureus.83449

**Published:** 2025-05-04

**Authors:** Rhuna Dhana, Yousef Aqel, Anurag Rawat, Aakash Mahato, Abdelaziz Maali Abusal, Nazish Munawar, Calvin R Wei, Adil Amin

**Affiliations:** 1 Paediatrics, Alder Hey Children's Hospital, Liverpool, GBR; 2 Medicine, Hamad Medical Corporation, Doha, QAT; 3 Interventional Cardiology, Himalayan Institute of Medical Sciences, Dehradun, IND; 4 Internal Medicine, Bishweshwar Prasad Koirala Institute of Health Sciences, Dharan, NPL; 5 Internal Medicine, Hamad General Hospital, Doha, QAT; 6 Internal Medicine, Alberta Health Services, Alberta, CAN; 7 Research and Development, Shing Huei Group, Taipei, TWN; 8 Cardiology, Pakistan Navy Station Shifa Hospital, Karachi, PAK

**Keywords:** cardiovascular outcomes, dapagliflozin, empagliflozin, sglt2 inhibitors, type 2 diabetes

## Abstract

Sodium-glucose co-transporter-2 (SGLT2) inhibitors have demonstrated significant cardiovascular benefits in patients with type 2 diabetes. However, head-to-head comparisons between dapagliflozin and empagliflozin, two widely prescribed SGLT2 inhibitors, remain limited. This meta-analysis aimed to directly compare the cardiovascular outcomes of these agents in patients with type 2 diabetes. We conducted a comprehensive literature search across multiple databases and included eight retrospective studies enrolling 280,617 patients (158,352 receiving empagliflozin and 122,265 receiving dapagliflozin). The primary outcome was major adverse cardiovascular events (MACE), with secondary outcomes including all-cause mortality, myocardial infarction, and stroke. Our pooled analysis revealed no significant difference in MACE risk between empagliflozin and dapagliflozin (RR: 1.04; 95% CI: 0.96 to 1.13). Similarly, no significant differences were observed for all-cause mortality (RR: 1.05; 95% CI: 0.96 to 1.15), myocardial infarction (RR: 1.04; 95% CI: 0.94 to 1.16), or stroke (RR: 1.00; 95% CI: 0.91 to 1.09). Subgroup analyses by gender, atherosclerotic cardiovascular disease, and chronic kidney disease status showed consistent results. However, in patients with heart failure, a trend toward reduced MACE risk was observed with empagliflozin (RR: 0.90; 95% CI: 0.82 to 1.00). Despite pharmacokinetic differences between these agents, our findings suggest comparable cardiovascular outcomes in patients with type 2 diabetes, with potentially enhanced benefits of empagliflozin in those with heart failure. However, due to lack of studies, this finding should be interpreted with caution. These results provide valuable insights for clinical decision-making when selecting SGLT2 inhibitors for cardiovascular risk reduction in diabetic patients. Further prospective studies are warranted to confirm these findings and explore potential mechanistic differences between these agents.

## Introduction and background

Diabetes mellitus (DM) continues to pose a significant global public health challenge due to its strong association with a wide range of complications, particularly atherosclerotic cardiovascular disease and heart failure [1-2]. Despite the availability of pharmacologic and lifestyle-based interventions, the risk of major cardiovascular events remains high, especially among individuals with type 2 diabetes in the primary prevention setting [3]. Sodium-glucose co-transporter-2 (SGLT2) inhibitors, a novel class of oral glucose-lowering agents, have shown substantial cardiovascular benefits compared to placebo and other glucose-lowering drugs (GLDs) in patients with type 2 diabetes [4-5]. These agents have gained increasing attention and are among the most extensively studied therapeutic options in recent years [6]. Among them, dapagliflozin and empagliflozin have become particularly prominent. 

A notable advantage of SGLT2 inhibitors is that their glucose-lowering mechanism is independent of insulin, achieved by inhibiting renal glucose reabsorption and enhancing its urinary excretion [7]. What distinguishes dapagliflozin and empagliflozin in diabetes care is their proven cardiovascular benefit. Multiple clinical trials have demonstrated their effectiveness in reducing cardiovascular risk in patients with diabetes and in those with heart failure with reduced ejection fraction [8]. 

Beyond glycemic regulation, SGLT2 inhibitors also positively influence cardiometabolic parameters, including reductions in body weight, blood pressure, and serum uric acid levels [9]. These benefits have contributed to their rising use in real-world clinical practice for managing type 2 diabetes [10]. However, differences in pharmacokinetic profiles among SGLT2 inhibitors should be noted. For instance, dapagliflozin is eliminated more slowly by the kidneys, allowing it to exert a longer-lasting effect, up to 18 hours post-dose, while empagliflozin’s efficacy diminishes significantly after 12 hours [11]. This prolonged activity with dapagliflozin, characterized by more sustained sodium excretion and osmotic diuresis, has been linked to more consistent 24-hour systolic blood pressure control and potentially lower cardiovascular risk compared to empagliflozin [12]. 

Although dapagliflozin and empagliflozin are among the most widely prescribed SGLT2 inhibitors globally, head-to-head comparisons are limited. Existing meta-analyses have highlighted challenges in directly comparing their cardiovascular effects due to differences in the patient populations enrolled across individual cardiovascular outcome trials, such as including subjects with or without heart failure [13-14]. New studies have been conducted since the recent meta-analysis was done [15]. While evidence supports the cardiovascular benefits of SGLT2 inhibitors as a drug class, variations in outcomes across individual agents remain. Therefore, this meta-analysis aims to directly compare the effectiveness of dapagliflozin versus empagliflozin in reducing cardiovascular events among patients with type 2 diabetes. 

## Review

Methodology

Literature Search 

A comprehensive literature search was conducted to identify relevant studies comparing the cardiovascular outcomes of dapagliflozin and empagliflozin in patients with type 2 diabetes mellitus. Electronic databases including PubMed, Embase, Web of Science, and Cochrane Library were systematically searched from inception to 15 April 2025 using a combination of Medical Subject Headings (MeSH) and free-text terms. The search strategy included terms such as "dapagliflozin", "empagliflozin", "SGLT2 inhibitors", "type 2 diabetes", "cardiovascular outcomes", "MACE", "myocardial infarction", "stroke", and "mortality". Additional studies were identified by manually screening the reference lists of relevant articles and reviews. No restrictions were placed on language or publication status. The search was independently carried out by two authors. Any disagreement between the two authors was resolved through consensus. 

Study Selection 

Studies were included if they met the following criteria: (1) observational studies or randomized controlled trials (RCTs) that directly compared dapagliflozin with empagliflozin; (2) enrolled patients diagnosed with type 2 diabetes mellitus; and (3) reported at least one cardiovascular outcome of interest, including major adverse cardiovascular events (MACE), all-cause mortality, myocardial infarction, or stroke. Studies were excluded if they were: (1) reviews, editorials, case reports, or conference abstracts; (2) not reporting comparative data between the two drugs; or (3) lacking sufficient data to extract relative risks (RR) or hazard ratios (HR). The selection process was performed independently by two reviewers, and discrepancies were resolved by consensus or consultation with a third reviewer. 

Data Extraction and Outcomes 

Two investigators independently extracted data using a standardized form. For each study, the following details were collected: first author, publication year, study design, country of origin, sample size, patient demographics (including age, sex, and baseline comorbidities), follow-up duration, and reported outcomes. The primary outcome assessed was the incidence of major adverse cardiovascular events (MACE), while secondary outcomes included all-cause mortality, myocardial infarction, and stroke. Data extraction was performed separately by both authors, with any discrepancies resolved through consensus. 

Quality Assessment 

The Newcastle-Ottawa Scale (NOS) was used to assess the methodological quality of the observational studies included. This scale evaluates studies across three domains: selection of study participants (0-4 points), comparability between groups (0-2 points), and outcome assessment (0-3 points). The overall NOS score ranges from 0 to 9, where a score of 7 or higher indicates high quality, 5-6 reflects moderate quality, and below 5 denotes low quality. Two reviewers conducted the quality assessment independently, and any disagreements were resolved through discussion. 

Data Analysis

Data analysis was performed using RevMan Version 5.4.1. To compare outcomes between empagliflozin and dapagliflozin, risk ratios (RR) with 95% confidence intervals were calculated for all endpoints using the random-effect model to deal with variations among the studies. A p-value less than 0.05 was considered statistically significant. Heterogeneity among studies was assessed using the I² statistic, with values of 25%, 50%, and 75% considered as low, moderate, and high heterogeneity, respectively. Forest plots were generated to visually represent the effect sizes across studies for each outcome. Comprehensive subgroup analyses were conducted to evaluate potential effect modifications by gender (male versus female), presence of heart failure, atherosclerotic cardiovascular disease (ASCVD) status, and chronic kidney disease (CKD) status. For each subgroup, risk ratios were calculated, and tests for interaction were performed to determine whether the treatment effect differed significantly between subgroups. We did not assess publication bias as the number included was less than 10.

Results

**Figure 1:**
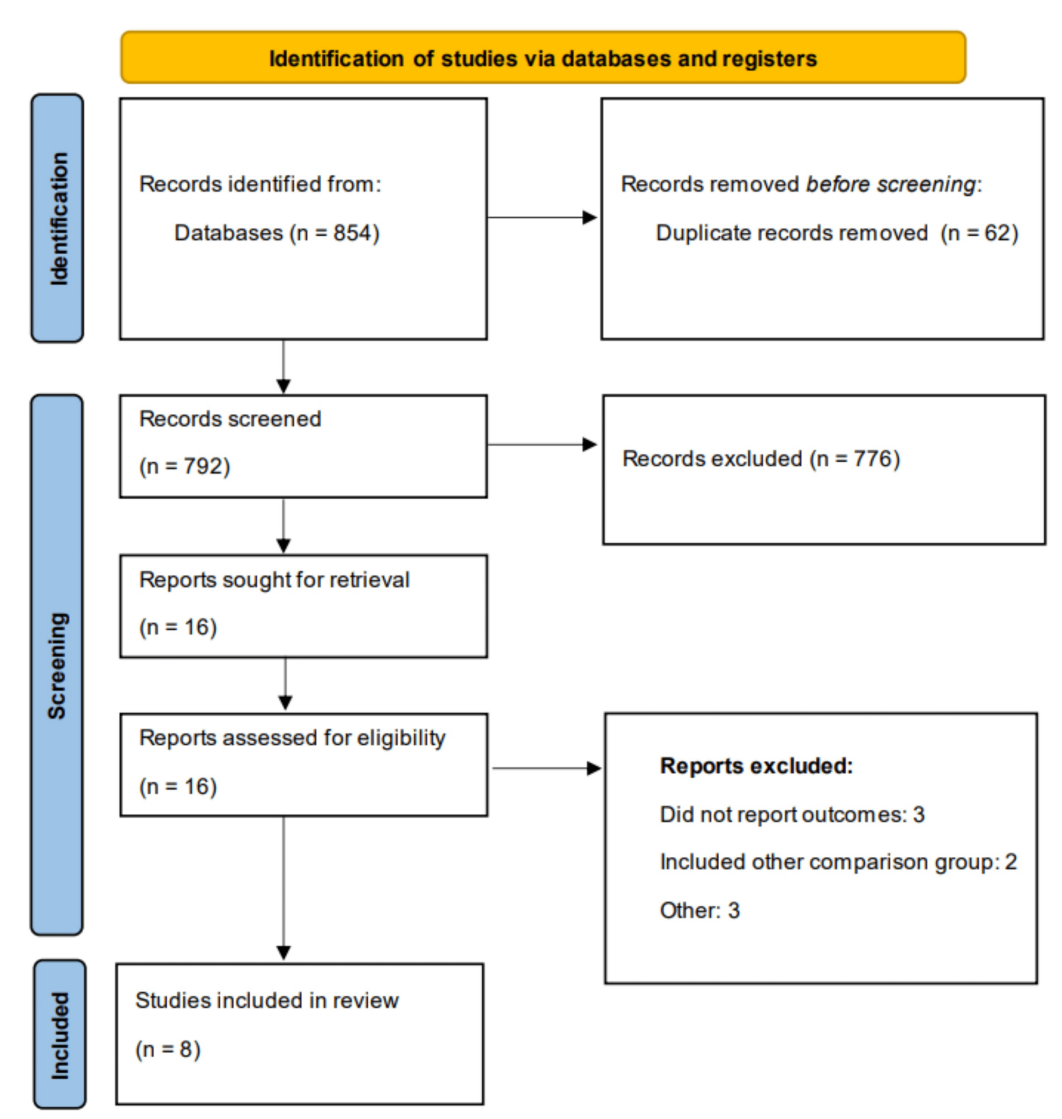
PRISMA flowchart (study selection process) PRISMA - Preferred Reporting Items for Systematic reviews and Meta-Analyses

The study selection process is shown in Figure 1. Initial database searching identified 854 studies. After removal of duplicates followed by initial and abstract screening, 16 studies were thoroughly reviewed based on the inclusion and exclusion criteria. From these, eight retrospective studies and their reports were included, enrolling a total of 280,617 subjects, of whom 158,352 received empagliflozin and 122,265 received dapagliflozin. Table 1 presents characteristics of the included studies. The follow-up duration ranged from 21.87 to 40.91 months. The mean age of participants, where reported, ranged from 52 to 62.4 years. Male participants constituted a substantial proportion of the study populations, with numbers ranging from 571 to 84,704 across studies where this information was available. Hypertension was a common comorbidity among participants, with prevalence ranging from 1079 to 84,361 patients in studies reporting this data. The sample sizes varied considerably across studies, with the largest cohorts reported by Bonnesen et al. (2024) and Lim et al. (2023), which included 57,276 and 145,504 patients, respectively. Park et al. (2022) had the smallest sample size with 1209 patients. Table 2 presents the quality assessment of included studies.

**Table 1:** Characteristics of included studies NR - not reported

Author	Year	Region	Study type	Sample size		Follow-up	Mean age (years)	Males (n)	Hypertension (n)
				Empagliflozin	Dapagliflozin				
Alhakak et al. [15]	2024	Denmark	Retrospective	40,003	21,028	37.2 months	62.6	38,205	NR
Bonnesen et al. [16]	2024	Denmark	Retrospective	36,670	20,606	37.2 Months	62.4	35,804	31,476
Kim et al. [17]	2024	Korea	Retrospective	537	537	40.91 months	57.35	703	610
Lim et al [18]	2022	Korea	Retrospective	921	921	37.2 months	56	1221	1079
Lim et al. [19]	2023	Korea	Retrospective	72,752	72,752	24.96 months	56.95	84,703	84,361
Park et al. [20]	2022	Korea	Retrospective	600	609	38.9 months	NR	NR	NR
Shao et al. [21]	2019	Taiwan	Retrospective	6869	5812	21.87 months	NR	4926	8324
Suzuki et al. [22]	2022	Japan	Retrospective	5302	4681	27.13 months	52	8230	5880

**Table 2:** Quality assessment of included studies Selection out of 4, Comparison out of 3, Outcome assessment out of 2

Author name	Selection	Group comparison	Outcome or exposure assessment	Overall
Alhakak et al. [15]	+++	++	++	7
Bonnesen et al. [16]	++++	++	+++	9
Kim et al. [17]	++++	++	+++	9
Lim et al. [18]	++++	++	++	8
Lim et al. [19]	++++	++	+++	9
Park et al. [20]	+++	+	++	6
Shao et al. [21]	+++	+	+++	7
Suzuki et al. [22]	+++	++	+++	8

Pooled Analysis of all Studies 

**Figure 2:**
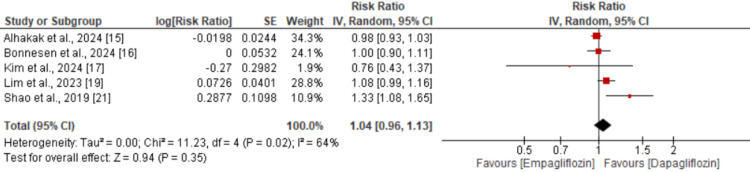
Comparison of MACE between two groups MACE - major adverse cardiovascular events Sources: references [15-17, 19, 21]

**Figure 3:**
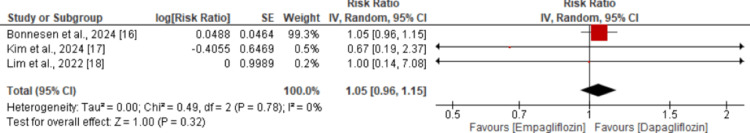
Comparison of all-cause mortality between two groups Sources: references [16-18]

**Figure 4:**
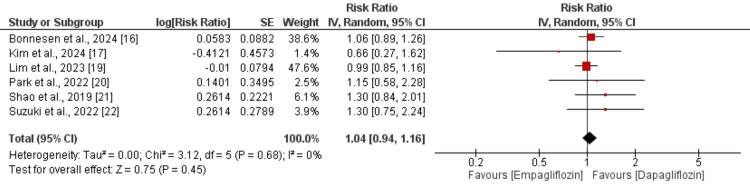
Comparison of myocardial infarction between two groups Sources: references [16-17, 19-22]

**Figure 5:**
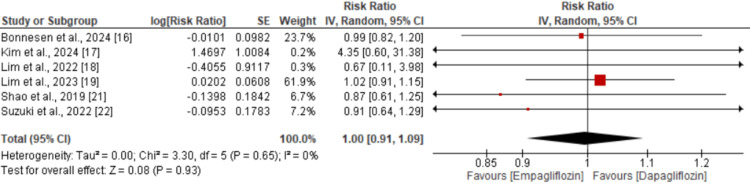
Comparison of stroke between two groups Sources: references [16-19, 21-22]

A total of five studies were included in the pooled analysis comparing the risk of major adverse cardiovascular events (MACE) between empagliflozin and dapagliflozin. As shown in Figure 2, there was no significant difference in the risk of MACE between the two drugs (RR: 1.04; 95% CI: 0.96 to 1.13). However, significant heterogeneity was observed across the studies (I² = 64%). Similarly, for all-cause mortality, no significant difference was found between empagliflozin and dapagliflozin (RR: 1.05; 95% CI: 0.96 to 1.15), as illustrated in Figure 3, with no significant heterogeneity reported (I² = 0%). Regarding the risk of myocardial infarction (MI), the pooled analysis also showed no significant difference between the two groups (RR: 1.04; 95% CI: 0.94 to 1.16), as shown in Figure 4, with no observed heterogeneity (I² = 0%). Lastly, a pooled analysis of six studies evaluating the risk of stroke demonstrated no significant difference between empagliflozin and dapagliflozin (RR: 1.00; 95% CI: 0.91 to 1.09), with no significant heterogeneity across studies (I² = 0%), as presented in Figure 5. 

Subgroup Analysis for MACE Outcomes 

Subgroup analyses were performed to evaluate the risk of major adverse cardiovascular events (MACE) between empagliflozin and dapagliflozin across different patient populations. No significant differences in MACE risk were observed between treatment groups when stratified by gender, with similar relative risks for males (RR: 1.01, 95% CI: 0.96 to 1.05, I² = 0%) and females (RR: 1.01, 95% CI: 0.88 to 1.16, I² = 33%). However, a significant interaction was detected in the heart failure subgroup (p = 0.04), where patients with heart failure showed a trend toward reduced MACE risk with empagliflozin compared to dapagliflozin (RR: 0.90, 95% CI: 0.82 to 1.00, I² = 19%), while no such trend was observed in patients without heart failure (RR: 1.02, 95% CI: 0.97 to 1.07, I² = 9%). In the atherosclerotic cardiovascular disease (ASCVD) subgroup, no significant differences were found between treatments, regardless of ASCVD status (without ASCVD: RR: 1.00, 95% CI: 0.94 to 1.05, I² = 0%; with ASCVD: RR: 0.97, 95% CI: 0.91 to 1.04, I² = 0%; p = 0.62 for subgroup differences). Similarly, chronic kidney disease (CKD) status did not significantly modify the comparative effectiveness of the two SGLT2 inhibitors (without CKD: RR: 1.00, 95% CI: 0.95 to 1.05, I² = 6%; with CKD: RR: 0.99, 95% CI: 0.77 to 1.27, I² = 29%; p = 0.92 for subgroup differences) (Table 3). 

**Table 3:** Subgroup analysis of MACE ASCVD - atherosclerotic cardiovascular disease; CKD - chronic kidney disease; RR - risk ratio; CI - confidence interval; MACE - major adverse cardiovascular events * Significant at p-value less than 0.05

Group	Categories	RR (95% CI)	I-squared	p-value of differences in groups
Gender	Male	1.01 (0.96 to 1.05)	0%	0.97
	Female	1.01 (0.88 to 1.16)	33%	
Heart failure	No	1.02 (0.97 to 1.07)	9%	0.04
	Yes	0.90 (0.82 to 1.00)	19%	
ASCVD	No	1.00 (0.94 to 1.05)	0%	0.62
	Yes	0.97 (0.91 to 1.04)	0%	
CKD	No	1.00 (0.95 to 1.05)	6%	0.92
	Yes	0.99 (0.77 to 1.27)	29%	

Discussion

In this meta-analysis, we compared cardiovascular outcomes between dapagliflozin and empagliflozin in patients with type 2 diabetes. Pooled analysis of eight studies showed that there is no comparison of any of the outcomes between MACE, myocardial infarction, all-cause mortality, and stroke between dapagliflozin and empagliflozin. Like earlier meta-analyses that found no clear differences among various SGLT2 inhibitors, this study did not demonstrate any significant difference between dapagliflozin and empagliflozin [13, 23]. 

A prior meta-analysis suggested that empagliflozin may offer a greater reduction in mortality compared to dapagliflozin [24]. Furthermore, another meta-analysis found that empagliflozin was more effective in lowering mortality and cardiovascular event rates [25]. Our findings are different because we included only those studies that directly compared the two treatment groups. Four retrospective meta-analyses included studies that directly compared dapagliflozin and empagliflozin in type 2 diabetes, and found no significant difference between myocardial infarction, stroke, heart failure, and cardiovascular mortality [14]. In the present meta-analysis, three of the included studies assessed all-cause mortality. None of the studies showed a significant difference in mortality between the two groups. 

The subgroup analysis revealed that the effect of dapagliflozin and empagliflozin on clinical outcomes is not significantly different in subjects with and without previous cardiovascular events. However, only studies that performed subgroup analysis were included in this study. The study performed by Lim et al. [18] enrolling subjects with a history of heart failure and any cardiovascular disease did not report any significant difference between all-cause mortality, cardiovascular mortality, and myocardial infarction between the two groups. These findings are consistent with our study and show that both of these SGLT2i inhibitors can benefit primary as well as secondary prevention of cardiovascular outcomes. However, the limited number of studies included in our subgroup analyses restricts the robustness of these findings. Further prospective studies with larger sample sizes and longer follow-up periods are needed to validate these subgroup-specific results and to determine whether certain patient populations might derive differential benefits from one agent over the other.

The cardioprotective benefits of empagliflozin primarily stem from its inhibition of NHE1 in heart muscle cells, which leads to decreased oxidative stress through AKT/NOS2 pathway modulation and enhanced mitochondrial function. These actions specifically target crucial aspects of heart failure with preserved ejection fraction (HFpEF) pathophysiology, including stiffening of the heart muscle and remodeling of the extracellular matrix [26]. In contrast, dapagliflozin shows effectiveness across various ejection fraction ranges, with its benefits linked to improved hemodynamics (lower preload/afterload) and metabolic changes (particularly utilization of ketone bodies) [27]. Importantly, both medications reduce systemic inflammation, a key contributor to atherosclerotic disease progression by suppressing the NLRP3 inflammasome and decreasing TGF-β1 levels [26]. This common anti-inflammatory effect likely explains why these drugs show comparable cardiovascular outcomes despite targeting different primary molecular mechanisms 

Study limitations 

Several limitations should be considered when interpreting the findings of this meta-analysis. First, all included studies were retrospective in design, which introduces potential selection bias and confounding factors that may not have been adequately controlled for despite statistical adjustments. Second, the significant heterogeneity observed in the MACE outcome analysis (I² = 64%) suggests variability in study methodologies, patient populations, or outcome definitions across studies. Third, the duration of follow-up, ranging from 21.87 to 40.91 months, may not have been sufficient to detect long-term differences between the two SGLT2 inhibitors. Fourth, information on medication adherence, dosing regimens, and concurrent medications was limited or unavailable in most studies, potentially influencing the observed outcomes. Fifth, our analysis primarily focused on cardiovascular outcomes and did not extensively evaluate safety profiles or other clinically relevant endpoints such as renal outcomes or glycemic control. Finally, the majority of included studies were conducted in Asian and European populations, potentially limiting the generalizability of our findings to other ethnic groups or healthcare systems. Future prospective randomized controlled trials directly comparing these agents are needed to address these limitations. 

## Conclusions

This meta-analysis comparing cardiovascular outcomes between dapagliflozin and empagliflozin in patients with type 2 diabetes found no significant differences in MACE, all-cause mortality, myocardial infarction, or stroke between the two SGLT2 inhibitors. However, subgroup analysis revealed a potential benefit of empagliflozin in patients with heart failure. However, there is a limited number of studies in subgroup analysis. Subgroup analysis findings need to be interpreted with caution. Despite pharmacokinetic differences between these agents, their overall cardiovascular effects appear comparable in the general diabetic population. These findings suggest that either medication can be effectively used for cardiovascular risk reduction in patients with type 2 diabetes, with treatment selection potentially guided by specific patient characteristics, particularly the presence of heart failure. Given the limitations of retrospective studies included in this meta-analysis, prospective randomized controlled trials directly comparing these agents are warranted to confirm these findings and explore potential mechanistic differences.
